# Pathological findings in the red fox (*Vulpes vulpes*), stone marten (*Martes foina*) and raccoon dog (*Nyctereutes procyonoides*), with special emphasis on infectious and zoonotic agents in Northern Germany

**DOI:** 10.1371/journal.pone.0175469

**Published:** 2017-04-11

**Authors:** Charlotte Lempp, Nicole Jungwirth, Miguel L. Grilo, Anja Reckendorf, Arlena Ulrich, Abbo van Neer, Rogier Bodewes, Vanessa M. Pfankuche, Christian Bauer, Albert D. M. E. Osterhaus, Wolfgang Baumgärtner, Ursula Siebert

**Affiliations:** 1 Department of Pathology, University of Veterinary Medicine Hannover, Hannover, Lower Saxony, Germany; 2 Center for Systems Neuroscience, Hannover, Germany; 3 Institute for Terrestrial and Aquatic Wildlife Research, University of Veterinary Medicine Hannover, Büsum, Schleswig-Holstein, Germany; 4 Department of Viroscience, The Erasmus University Medical Center, Rotterdam, the Netherlands; 5 Institute of Parasitology, Justus Liebig University Giessen, Giessen, Germany; 6 Research Center for Emerging Infections and Zoonoses, University of Veterinary Medicine Hannover, Hannover, Lower Saxony, Germany; University of Pretoria, SOUTH AFRICA

## Abstract

Anthropogenic landscape changes contributed to the reduction of availability of habitats to wild animals. Hence, the presence of wild terrestrial carnivores in urban and peri-urban sites has increased considerably over the years implying an increased risk of interspecies spillover of infectious diseases and the transmission of zoonoses. The present study provides a detailed characterisation of the health status of the red fox (*Vulpes vulpes*), stone marten (*Martes foina*) and raccoon dog (*Nyctereutes procyonoides*) in their natural rural and peri-urban habitats in Schleswig-Holstein, Germany between November 2013 and January 2016 with focus on zoonoses and infectious diseases that are potentially threatening to other wildlife or domestic animal species. 79 red foxes, 17 stone martens and 10 raccoon dogs were collected from traps or hunts. In order to detect morphological changes and potential infectious diseases, necropsy and pathohistological work-up was performed. Additionally, in selected animals immunohistochemistry (influenza A virus, parvovirus, feline leukemia virus, Borna disease virus, tick-borne encephalitis, canine adenovirus, *Neospora caninum*, *Toxoplasma gondii and Listeria monocytogenes)*, next-generation sequencing, polymerase chain reaction (fox circovirus) and serum-neutralisation analysis (canine distemper virus) were performed. Furthermore, all animals were screened for fox rabies virus (immunofluorescence), canine distemper virus (immunohistochemistry) and Aujeszky’s disease (virus cultivation). The most important findings included encephalitis (n = 16) and pneumonia (n = 20). None of the investigations revealed a specific cause for the observed morphological alterations except for one animal with an elevated serum titer of 1:160 for canine distemper. Animals displayed macroscopically and/or histopathologically detectable infections with parasites, including *Taenia sp*., *Toxocara sp*. and *Alaria alata*. In summary, wildlife predators carry zoonotic parasitic disease and suffer from inflammatory diseases of yet unknown etiology, possibly bearing infectious potential for other animal species and humans. This study highlights the value of monitoring terrestrial wildlife following the “One Health” notion, to estimate the incidence and the possible spread of zoonotic pathogens and to avoid animal to animal spillover as well as transmission to humans.

## Introduction

Wild carnivores have increasingly entered human habitats within the past decades, mainly due to anthropogenic habitat alterations as a result of the growing human population [1–4]. The unintentional convergence of human and wildlife populations has been enhanced by several predator species which readily learn to take advantage of easily accessible food sources in urban and peri-urban habitats [2, 4]. One of these highly adaptable predators is the red fox (*Vulpes vulpes*), that is the most widespread wild terrestrial carnivore in European countries like Germany, Estonia, Great Britain, and Switzerland [5–8]. Moreover, the red fox was the first wild carnivore which was subjected to scientific investigation, due to its increasing occurrence in British cities in the 1930s [7]. Due to its high adaptability and an area-wide rabies eradication programme using oral vaccination in the 1980s, the red fox population has considerably increased in Germany [9]. Another common wild predator in Germany, with stable population numbers since the early 1980s, is the stone marten (Martes foina, [9, 10]). In addition, the raccoon dog (*Nyctereutes procyonoides*), an invasive species, imported from Asia as a fur-bearing animal to Eastern Europe has expanded its range throughout Germany since the 1960s [11]. All these species have started to invade human habitats due to the benefits of urbanisation and now live in close contact with humans, exposing them to a number of potentially zoonotic diseases. To prevent and control the spillover of potentially zoonotic and/or infectious pathogens, new interdisciplinary collaborations like the “One Health” concept have been established [12]. While the fox rabies virus (*Rabies lyssavirus)* has been eradicated in Germany [13], other infectious diseases of wild carnivores, including zoonotic parasitoses such as *Echinococcus multilocularis*, viral (e.g. canine distemper virus (CDV) or avian influenza virus and bacterial (e.g. *Listeria monocytogenes*) pathogens, still represent a potential threat to humans who come into contact with those predators or their excretions [2, 14, 15].

Parasitic diseases of wild carnivores harbouring zoonotic potential include *Echinococcus multilocularis*, *Taenia* spp., *Alaria alata*, *Uncinaria stenocephala*, *Ancylostoma caninum*, *Toxocara canis* and *Toxascaris leonina*, causing a variety of diseases in humans mainly due to migrating larvae [2, 16]. Additionally, *Toxoplasma gondii* and *Neospora caninum* represent parasitic diseases with a considerable economic impact within ruminants due to foetal mortality caused by intrauterine infections as well as severe neurological deficits in dogs. Further, Toxoplasmosis is known to cause disease in humans not only in pregnancies but also in immunocompetent adults in which some strains lead to severe pneumonia and encephalitis [17, 18]. *Neospora caninum* has additionally been described as an opportunistic pathogen of HIV-infected patients and patients with neurological disorders [19–21]. Another well known zoonosis carried by wild carnivores is echinococcosis, with two important types for humans. These are, according to their wide geographical distribution as well as their medical and economic impact, cystic echinococcosis caused by *Echinococcus granulosus* and alveolar echinococcosis induced by *Echinococcus multilocularis* [22, 23]. *Echinococcus granulosus* plays a minor role in wild carnivores in Western and Central Europe. In contrast, a major role for the transmission of *Echinococcus multilocularis* is attributed to the red fox, the raccoon dog and the Arctic fox as definitive hosts [23]. Rodents are the intermediate hosts within the sylvatic cycle [23]. Domestic dogs can function as susceptible definitive hosts by becoming infected via ingestion of wild intermediate hosts [24]. Infections of humans usually occur through ingestion of infectious eggs and can lead to larval encystation and proliferation mainly in the liver with a subsequent spread via blood and lymph vessels to other organs [22, 23].The wide variety of known zoonotic diseases in wild carnivores highlights the necessity of periodical monitoring of the wildlife population to allow a proper assessment of the current health status and parasitic burden in predators and their role in zoonotic and infectious disease transmission and potential spillover.

In addition, consideration should be given to morbilliviruses, with special emphasis on canine distemper virus (CDV). This paramyxovirus has increasingly spread among wildlife populations including carnivorous species like red foxes, raccoons, raccoon dogs or minks, but also infects marine mammals [25–28]. This has been shown in massive outbreaks with high mortality rates among the seal population in 1988, 2000, 2001 and 2002 in Europe, caused by CDV and the closely related but genetically different phocine distemper virus (PDV) in the North Sea, East Greenland Coast, Caspian Sea and Lake Baikal, respectively [25, 29–31]. In cases where CDV has been demonstrated to be the causative agent, it has been assumed that terrestrial carnivores might have caused a spillover towards the marine population [25]. This phenomenon has already been described as a possible route of infection between different carnivore species as well as within non-human primates [29, 32, 33].

In addition, the recent outbreak of the highly pathogenic avian influenza A virus in harbour seals (H10N7) in the coastal waters of Schleswig-Holstein, Germany resulted in numerous dead seals [34]. In this context, other investigations have demonstrated a natural infection with avian influenza virus (H5N1) in a stone marten [35] as well as the possible susceptibility of red foxes to become infected by eating infected bird carcasses [36].

These events highlight the necessity of monitoring wild carnivores' susceptibility to CDV and avian influenza A virus to predict possible epidemic spread among these populations, as well as possible transmission to domestic dogs or cats.

Moreover, the introduction of new diagnostic tools including molecular methods, such as next generation sequencing (NGS), enables the detection of potentially unknown infectious diseases, especially viral infections in animals displaying lesions of unknown etiology. This approach has led to a number of newly detected viruses in various animal species in recent years [37–40] and might be of great interest for detecting potentially zoonotic and infectious diseases in wildlife species.

This scientific approach of an overall contemplation of animal diseases in relation to the urban wildlife-human interface follows the idea of “One Health” notion, aiming at a conceived observation of the current health status of wildlife animals in Northern Germany to allow early detection of possible threats to the wildlife population, humans and domestic animal species.

## Materials and methods

### Investigated animals and histology

A total of 79 foxes, 17 stone martens and ten raccoon dogs were examined (S1 Table). Of the 79 foxes, 15 were juveniles (deciduous teeth) and 64 were adults (permanent teeth) with a gender distribution of 36 males and 43 females. Of the collected stone martens, 15 were adults and two were juveniles, six of them were males and 11 were females. Similarly, eight raccoon dogs showed permanent teeth (adults) and two showed deciduous teeth (juveniles), with 3 males and 7 females.

**Fig 1:**
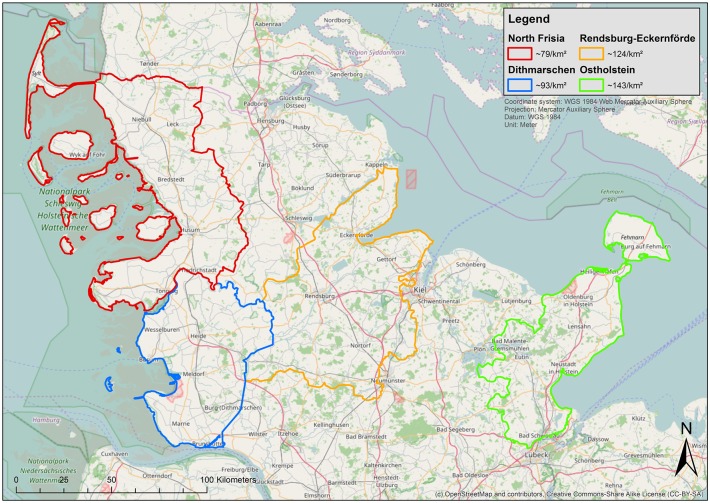
Overview of all areas in natural habitats of Schleswig-Holstein from which animals were captured. In the legend, an average human population density (inhabitants per square kilometre) is given for the respective district including North Frisia (red), Redsburg Eckenförde (yellow), Dithmarsch (blue) and Ostholstein (green; Statistical Office for Hamburg and Schleswig-Holstein, 2015, [41]).

Animals included in this study were either trapped and euthanised, hunted during the licenced hunting season or found dead. These hunts aim at reducing predator pressure on wildlife in the state of Schleswig-Holstein. Selection of animals was random. The animals were collected between November 2013 and January 2016 in North Frisia, Dithmarschen, Ostholstein and Rendsburg-Eckernförde. These are districts of the state of Schleswig-Holstein (Fig 1).

Of the 79 foxes, 27 (11 males, 16 females) were caught in a concrete pipe trap, and 15 (six males, nine females) of the 17 stone martens were caught in a wooden box trap. All of the animals were adults, except for a juvenile male red fox and a juvenile female stone marten. The number of trapped animals varied with the year of collection (2013—eight red foxes, eight stone martens; 2014–17 red foxes, four stone martens; 2015 –two red foxes, three stone martens). The majority of the animals (21 red foxes, 13 stone martens) were trapped during the control season, (between October and February), while the other animals (two red foxes, one stone marten) were trapped between March and August. None of the animals were reported to show any clinical signs of disease or abnormal behaviour. Trapped animals that underwent general clinical examination did not display signs of disease either. Trapped animals were anaesthetised intra-muscularly using a combination of medetomidine (fox: 0.1mL/2kg; stone marten 0.1 mL/1kg; Cepedor^®^, CP-Pharma, Burgdorf, Germany) and ketamin (fox: 0.1mL/1kg; stone marten: 0.1mL/1g; Ketamin^®^, CP-Pharma, Burgdorf, Germany). Subsequently, euthanasia was performed using embutramide, mebezonium iodide, tetracaine hydrochloride (T61^®^, MSD, Unterschleissheim, Germany), intracardially (1.5 mL/kg). Remaining foxes, stone martens and all raccoon dogs were either killed by shooting during legal hunts or were found dead (S1 Table). Animal trapping, handling and euthanasia were conducted in accordance with the German Animal Welfare Law and all experiments were approved by the official agency for animal experiments in Schleswig-Holstein, The Ministry of Energy, Agriculture, the Environment and Rural Areas (MELUR) under the permit V242-7224.121–19.

Afterwards, all animals were sent to the Department of Pathology at the University of Veterinary Medicine Hannover, Germany, either refrigerated or deep frozen and subsequently examined during a complete necropsy.

Gross lesions were frequently related to extensive traumatic lesions of the musculoskeletal system caused by the gunshots and included muscle and organ lacerations, bone fractures and acute haemorrhages. These lesions were not included in the evaluation of the health status.

### Histopathology

Organ samples taken from each animal included central and peripheral nervous system, upper and lower respiratory tract, gastrointestinal tract (oesophagus, stomach, small and large intestine and rectum, including anal sacs), liver, spleen, pancreas, urogenital tract (kidneys, urinary bladder, gonads, prostate gland), heart, lymphatic organs (spleen, lymph nodes, thymus if present), bone marrow, skeletal muscle, endocrine organs (thyroid glands, andrenal glands, hypophysis), skin and eye. Samples from each organ were collected and fixed in 10% neutral-buffered formalin for 24–48 hours, dehydrated, embedded in paraffin wax and sections were stained with hematoxylin-eosin (HE). Tissues with granulomatous inflammation were examined by Ziehl-Neelsen staining for visualising *Encephalitozoon cuniculi* organisms and acid-fast bacteria as described by Haist [42]. Selected cases with suspected mineralisation of tissues were additionally examined by von-Kossa staining.

### Testing for fox rabies and Aujeszky’s disease

In order to rule out fox rabies virus and Aujeszky’s disease virus, samples were collected from each animal and examined at the Department of Consumer and Food Safety of Lower-Saxony, Hannover, Germany. The hippocampus was examined by immunofluorescence for the presence of rabies virus antigen and samples from the brain stem, olfactory bulb, lumbar spinal cord and trigeminal ganglion were tested for the presence of Aujeszky’s disease virus by cultivation.

### Immunohistochemistry

Immunohistochemical investigation was performed in accordance with standard procedures as previously described [43–48]. Sections of archived material from immuno-positive animals were used as adequate positive controls. Briefly, antigen retrieval was performed in citrate buffer (pH 6.0, 20 min, 95°C). Subsequently, endogenous peroxidase activity was blocked using 0.5% hydrogen peroxide in 70% ethanol for 30 min. Nonspecific protein binding was blocked using normal goat serum (20% in phosphate buffered saline, 30 min). Primary antibodies as listed in Table 1 were added and incubated for 2 hours. To receive appropriate negative controls, primary antibodies were replaced by ascites fluid from non-immunised Balb/cJ mice or rabbit control serum, both diluted in phosphate buffered saline with 1% bovine serum albumin (BSA). Following this, secondary antibodies were added and incubated for 30 min. The avidin-biotin complex (ABC) method (VECTASTAINING Elite ABC Kit, Vector Laboratories, Burlingame, California, USA) was undertaken according to the manufacturer’s instructions. 3, 3′-diaminobenzidine-tetrahydrochloride (DAB, Sigma-Aldrich, Munich, Germany) was used as chromogen. Sections were counter-stained with Mayer’s hematoxylin. Routinely, a spectrum of organs from each animal, including cerebellum, lungs, stomach, urinary bladder, spleen and lymph nodes *(Lymphonodi mesenteriales*, *tracheobrochiales*, *poplitei and retropharyngeales)*, as well as inflamed areas, were used for detecting distemper virus antigen. In addition, lungs from foxes and stone martens killed at the North Sea coast during the seal influenza outbreak in 2014/2015 [34] were investigated using an influenza A virus specific antibody. All animals with inflammatory CNS lesions were further investigated using a series of antibodies and further methods, as summarised in Tables 1 and 2.

**Table 1:** Antibodies used for detecting viral and protozoal antigens.

Infectious agent	Clone	Specificity	Supplier/Reference	Clonality and source	Dilution
Canine distemper virus	anti-canine distemper nucleoprotein antibody (#25)	Nucleoprotein	Prof. Örvell ^a^/Baumgärtner [49]	Polyclonal rabbit	1:2000
Influenza A	HB65	Nucleoprotein	European Veterinary Laboratory ^b^/van Baalen [50]	Monoclonal mouse	1:200
Parvovirus	CPV1-2A1	Unknown	Custom Monoclonal Antibodies International ^c^/Kipar [51]	Monoclonal mouse	1:200
Feline leukaemia virus	C11D8	Glycoprotein 70	Custom Monoclonal Antibodies International ^d^/Elder [52]	Monoclonal mouse	1:200
Borna disease virus	Bo18	Nucleoprotein	Dr. Herzog ^d^	Monoclonal mouse	1:500
Tick-borne encephalitis	K-D-3.BA	Not stated	Prof. Holzmann^f^/Weissenböck [53]	Polyclonal rabbit	1:300
Toxoplasma gondii		Not stated	Quartett ^g^/Klein [54], Brack [55]	Polyclonal rabbit	1:75
Neospora caninum		Not stated	Dr. Schares ^h^	Monoclonal mouse	1:2
Canine adenovirus 1		The whole CAV1 tribe “Mirandola”	Chemicon ^i^	Monoclonal mouse	1:250
Listeria monocytogenes 1,4		The whole organism	Difco Laboratories ^j^	Polyclonal rabbit	1:2000

CPV, canine parvo virus; Bo, Borna disease;

^a^ Central Microbiological Laboratory of Stockholm county, Stockholm Council, Sweden;

^b^ Woerden, the Netherlands;

^C^ Sacramento, California, USA

^d^Institute of Virology, Justus Liebig University, Giessen, Germany;

^e^ Vienna, Austria;

^f^ Berlin, Germany;

^g^ Munich, Germany;

^h^ Institute of Epidemiology, Friedrich-Loeffler-Institut, Federal Research Institute for Animal Health, Greifswald—Insel Riems, Germany;

^i^ Hamburg, Germany;

^j^ Detroit, USA

**Table 2:** Summary of all performed investigations including animal species, tissues investigated and method applied.

Method	Animals tested			Results			Used material
	Red fox (n = 79)	Stone marten (n = 17)	Raccoon dog (n = 10)	Red fox (n = 79)	Stone marten (n = 17)	Raccoon dog (n = 10)	
**IHC**							FFPE^a^
CDV	79	17	10	-	-	-	
FeLv	9	4	3	-	-	-	
Parvovirus	9	4	3	-	-	-	
Borna disease	9	4	3	-	-	-	
Toxopasma gondii	9	4	3	-	-	-	
Influenza A	17	5	3	-	-	-	
Tick-borne encephalitis	9	4	3	-	-	-	
Neospora caninum	9	4	3	-	-	-	
Listeria monocytogenes	9	4	3	-	-	-	
Canines adenovirus 1	9	4	3	-	-	-	
**PCR**							FFPE^b^
fox circovirus DNA	9	n.t.	n.t.	-	n.t.	n.t	
**SNT**	16	n.t.	2	+	n.t.	-	Serum
**NGS**	5	1	n.t.	2+	-	n.t	Native material^c^
**IF**							
Fox rabies virus	79	17	10	-	-	-	Native material^d^
**Virus cultivation**							Native material^e^
Herpes suis (Aujeszky’s disease)	79	17	10	-	-	-	
**Ziehl-Neelsen staining**							FFPE^f^
acid-fast bacteria/ Enzephalitozoon cuniculi	1	2	4	-	-	-	

FFPE, Formalin-fixed paraffin-embedded; IHC, Immunohistochemisty; PCR, Polymerase chain reaction; SNT, Serum neutralization test; NGS, Next generation sequencing; IF, Immunofluorescence; Native material (fresh non formalin-fixated material);

^a^ Brain tissue or lung tissue depending on the localisation of the lesion;

^b^ brain tissue;

^c^cerebrum;

^d^ hippocampus;

^e^ brain stem, olfactory bulb, lumbar spinal cord & trigeminal ganglion;

^f^ lung- or brain tissue; -, negative; +, positive; n.t., not tested

### Viral metagenomics and RT-PCR

Brain tissue samples of six different animals (five foxes and one stone marten; S2 Table) were analysed by sequence-independent RNA and DNA virus screening and next-generation sequencing (NGS) using the 454 Sequencing platform (GS Junior, Roche) as previously described [56, 57]. Obtained reads were analysed by BLASTN and BLASTX with a bioinformatics virus discovery pipeline using standard parameters as previously described [58]. The European Nucleotide Archive Study accession number of this study is PRJEB19187, with secondary study accession number ERP021170.

Foxes with non-suppurative encephalitis were tested for the presence of fox circovirus DNA using real-time quantitative polymerase chain reaction (PCR, [59]). DNA was isolated from formalin-fixed paraffin embedded tissue (FFPE) according to the manufacturer´s protocol (QIAamp DNA FFPE Tissue Kit; Qiagen, Venlo, the Netherlands) as described previously [60]. Briefly, eight 7 μm thick sections of FFPE tissue were de-paraffinised, lysed under denaturing conditions with proteinase K and incubated at 90°C to reverse formalin crosslinking. DNA was purified using a silica gel-based membrane. Real- time quantitative PCR (RT-qPCR) was performed as formerly described with minor variations using fox circovirus specific primers VS756 (5′-TCCGAGATAGCC GGCGTGGTA-3′) and VS757 (5′-CCCGGCCACAGATCAAGTACTTA-3′, [58]), the Mx3005P QPCR System (Agilent Technologies, Santa Clara, USA) and pre-diluted SYBR-Green qPCR master mix (Brilliant III Ultra-Fast SYBR Green QPCR Master Mix; Agilent Technologies, Santa Clara, USA). 50ng/μl of isolated DNA per sample served as a template and a run was performed according to the manufacturer´s protocol with minor variations under the following conditions: 95°C for 3min; 95°C for 5 sec and 60°C for 20 sec repeated 35 times; 95°C for 1 min, 55°C for 30 sec and 95°C for 30 sec. A no template control was included in the run. The isolated fox DNA served as positive control, using canine Glycerinaldehyd-3-phosphat-Dehydrogenase (GAPDH) specific primers (forw: 5′-GTCATCAACGGGAAGTCCATCTC-3′; rev: 5′-AACATACTCAGCACCAGCATCAC-3′) as well as DNA from a pig suffering from porcine circovirus 2 (PCV-2) using PCV-2 specific primers (forw: 5´-GCACCCTGTAACGTTTGTCA-3´; rev: 5´ATTTTCCCGCTCACTTTCAA3´).

### Serum neutralisation test

Serum samples from 30 animals (16 foxes, 12 stone martens and two raccoon dogs) were examined for the presence of anti-CDV neutralising antibodies. A standard test was performed as previously described [61, 62]. Sera were heat inactivated for 30 minutes and prepared in a starting dilution, 1:10 in medium within 96-well microtitration plates (Nunc, Roskilde, Denmark). A twofold serum dilution of 50 μl was tested in quadruplicate. Subsequently, 50 μl including a 100 media tissue culture infection dose of the Onderstepoort CDV strain (Ond-CDV; kindly provided by A.E. Metzler, Institute of Virology, University Zurich, Switzerland, [63]) was added to each well and incubated for 1 hour at 37°C. Afterwards, 100 μl of a Vero cell suspension were added and the plates were incubated under standard culture conditions for 3 to 5 days. Following incubation, wells were examined for the neutralising capacity of the sera which is detected by inhibiting the cytopathic effect induced by the presence of the Ond-CDV [61]. Further calculation of the neutralisation titer was performed according to Reed and Muench [64].

## Results

### Distribution of lesions and immunohistochemical results

**Fig 2:**
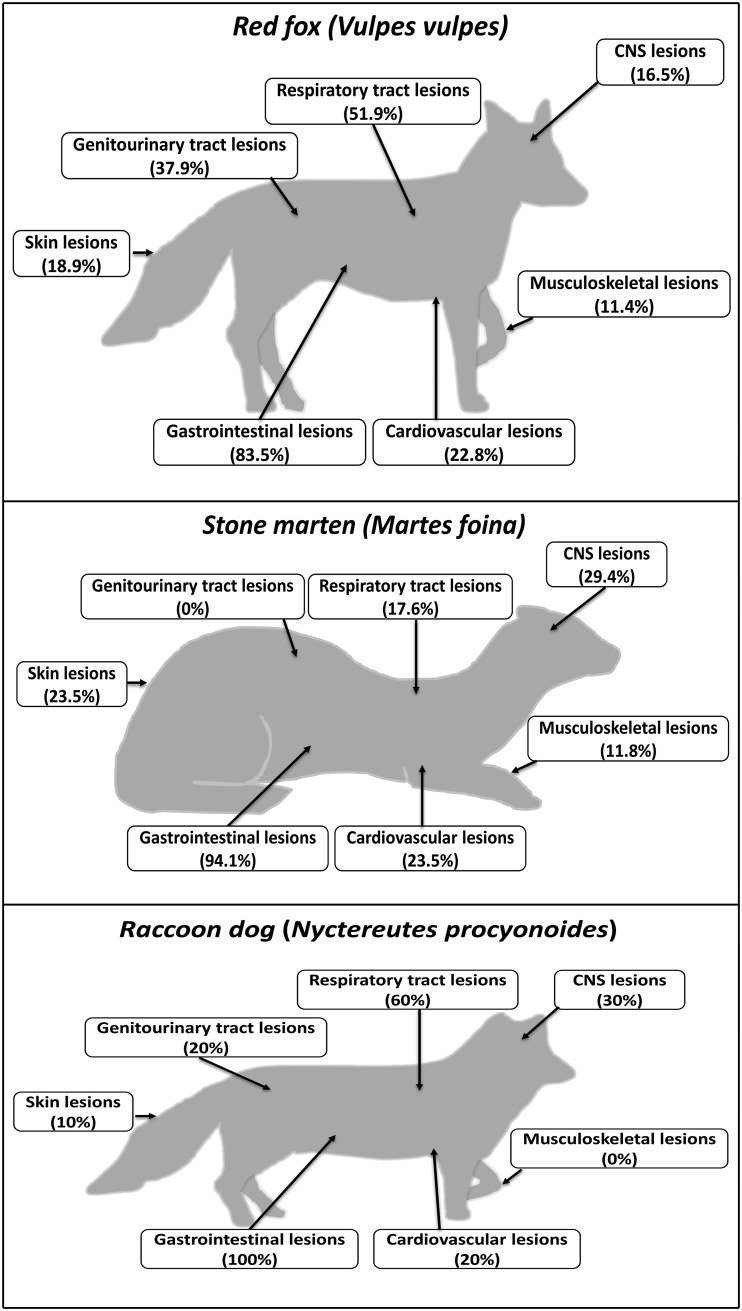
Distribution of lesions in the organ systems of foxes, stone martens and raccoon dogs in percentage of animals affected. CNS = central nervous system.

Lesions identified in the different species are summarised in Fig 2 listed by organ systems. All investigated animals and relevant morphological alterations are listed in S1 Table.

#### Central Nervous System (CNS)

**Fig 3:**
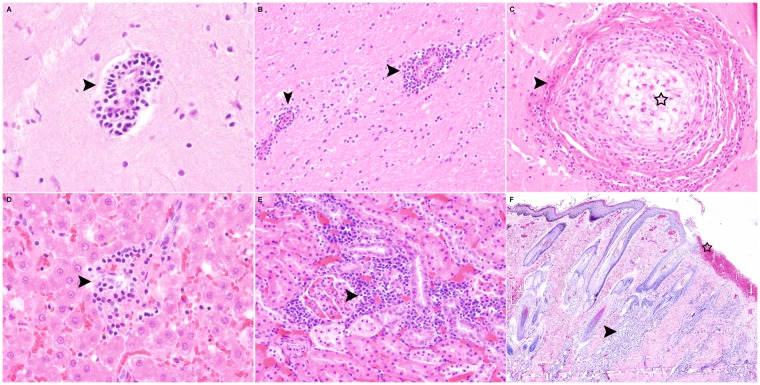
Inflammatory lesions in wild carnivores. (**A, B)** Cerebrum. Encephalitis in a fox (**A**; HE x400) and a stone marten (**B**; HE x200), displaying mild to moderate, perivascular, lymphohistiocytic and plasmacytic infiltration (arrowheads). **(C)** Cerebral cortex. Granulomatous encephalitis in a raccoon dog. Focal granulomatous encephalitis with a necrotic centre (asterisk) and mild compression of the surrounding neuroparenchyma (arrowhead) are present (HE x200). **(D)** Liver. Hepatitis in a stone marten. Mild, multifocal, mostly periportal, lymphohistiocytic infiltration (arrowhead; HE x400). **(**E) Kidney. Nephritis in a fox. The interstitium is expanded by moderate numbers of multifocally distributed, lymphoplasmacytic and histiocytic infiltrates (arrowhead; HE x200). **(F)** Skin. Dermatitis in a fox. Focally, there is extensive ulceration with acute haemorrhage (asterisk). The underlying dermis shows multifocal, perivascular and periadnexal chronic suppurative inflammation (arrowhead; x40). HE, hematoxylin and eosin.

In nine (11.4%) foxes, four (23.5%) stone martens and three (30%) raccoon dogs, a mild to moderate, multifocal, mostly lymphohistiocytic perivascular encephalitis with few plasma cells (Fig 3), partly with involvement of the meninges (7/ 16 animals; 44%) was observed. One of the raccoon dogs had a granulomatous encephalitis (Fig 3). These cases were further investigated by immunohistochemistry and were negative for canine distemper virus (CDV) antigen, as well as for Borna virus, feline leukaemia virus (FeLV), parvovirus, tick-borne encephalitis (TBE) virus, influenza A virus, canine adenovirus (CAV1), *Listeria monocytogenes*, *Neospora caninum* and *Toxoplasma gondii*. Ziehl-Neelsen staining for visualising *Encephalitozoon cuniculi* spores was also negative. In addition, six of these cases underwent analysis by next generation sequencing; however, no specific virus fragments were identified. Additional morphological findings in the CNS included single cases of white matter vacuolisation, meningeal mineralisation and mild gliosis.

#### Respiratory system

**Fig 4:**
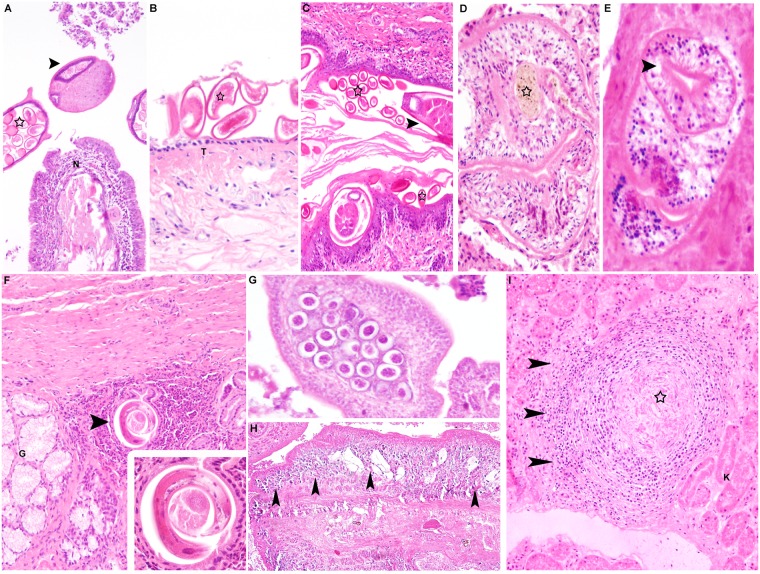
Parasites in tissue sections. Manifestation of *Capillaria* infection in various tissues in foxes. **(A)** Nasal mucosa (N), fox. Mild, diffuse, lymphoplasmahistiocytic inflammation. Within the lumen of the nasal cavity, there are adult nematodes (arrowhead) with a prominent stichosome and a large number of oval, embryonated eggs (star), morphologically consistent with *Capillaria* sp (HE x100). **(B)** Trachea, fox. *Capillaria* eggs (star) are also present on the surface of the tracheal mucosa (T; HE x200). **(C)** Anal sac, fox. The epithelium is markedly thickened and expanded by a large number of nematode stages, adult nematodes (arrowhead) as well as nematode eggs (asterisk) embedded in the stratum corneum (HE x100). Manifestation of trematode infection **(D, E)** Lung, raccoon dog. Single adult trematodes with brownish pigment (**D**; asterisk) and a sucker (**E**, arrowhead) resembling *Alaria alata*. There is a lack of pronounced inflammatory response to these parasites (HE x200). **(F)** Stomach, raccoon dog. Focal, granulomatous inflammation beneath gastric glands (G) with an intralesional nematode parasite (arrow), morphologically consistent with *Ollulanus tricuspis* (HE x100). Inset: Higher magnification of the nematode (HE x400). **(G, H)** Intestine, fox. Presence of cestodes in the intestinal lumen (HE x200). **(H**) Note numerous calcareous corpuscules in the cestode organism resembling *Taenia sp*. (arrowheads; HE x40). **(I)** Kidney, raccoon dog. Granulomatous nephritis. Centrally, there is evidence of a necrotic parasite, presumably larval stages of *Toxocara* (asterisk; HE x100). HE, hematoxylin and eosin.

Significant lesions of the respiratory system were generally rare, mild and characterised by inflammatory changes. 5 out of 79 foxes (6%) exhibited *Capillaria* sp. eggs within the nasal and/or tracheal mucosa (Fig 4), accompanied by minimal, lymphohistiocytic and eosinophilic inflammation. A mild pneumonia and/or bronchopneumonia, with variable infiltration by lymphocytes, plasma cells, macrophages and occasionally eosinophils was present in 16 (20.3%) foxes, one (5.9%) stone marten and in three (30%) raccoon dogs. Granulomatous pneumonia was present in five foxes (6%), two stone martens (11%) and three raccoon dogs (30%). Ziehl-Neelsen staining was negative and no causative agents were identified within the granulomatous lesions. Also, immunohistochemistry for influenza A virus was negative in eight foxes (10%) and one stone marten (5.9%) with interstitial pneumonia. In three foxes (3.7%) and four raccoon dogs (40%) *Alaria alata mesocercaria* (Fig 4) were present without associated lesions. Other findings in the respiratory tract were focal osseous metaplasia, alveolar histiocytosis and multifocal lipid pneumonia in one animal each.

#### Cardiovascular system

Cardiovascular lesions were only present in foxes. Two of the examined animals (mature red foxes, according to the teeth condition) displayed myxoid degeneration of the valvular stroma at the atrioventricular valves indicating valvular endocardiosis. In addition, one fox showed mild lymphohistiocytic myocarditis and focal myocardial mineralisation was observed in another animal. Moreover, dextroposition of the aorta was observed in one fox. An aortic aneurysm was detected in another animal. In one marten, intramyocardial *Sarcocystis* cysts were observed.

#### Digestive tract

Most digestive tract lesions were locally circumscribed and of a low degree of severity. Ulcerative and granulomatous glossitis, partly with intralesional foreign material resembling plant material and hair fragments were present in eight animals (five foxes (6.3%); one stone marten (5.9%) and two raccoon dogs (20%)). One animal showed loss of multiple teeth with alveolitis and gingivitis. Ten foxes had mild, bilateral, suppurative and erosive inflammation of the tonsils. One juvenile fox suffered from diffuse, catarrhal enteritis. Otherwise, there were no significant inflammatory lesions present in stomachs or intestines of any of the examined animals. Additionally, histological evidence of metazoan parasites including *Taenia sp*. and *Toxocara sp*. were found in the intestine of 13 foxes and one raccoon dog. Furthermore, nematode stages, most likely of *Ollulanus tricuspis* (Fig 4), could be identified within the stomach of one raccoon dog. A mild to moderate, lymphohistiocytic and suppurative inflammation and moderate hyperkeratosis of the anal sac epithelium, partly accompanied by nematode eggs and rarely adult stages of *Capillaria* sp. in the epithelium of the anal sac (Fig 4) were present in 25 foxes, seven stone martens and four raccoon dogs. Minimal to mild hepatitis (Fig 3) was present in 26 of 106 animals with variable, mostly lymphohistiocytic and periportal, necrotising or granulomatous character.

#### Genitourinary tract

The most consistent finding within the urinary tract was chronic interstitial nephritis (Fig 3), mostly dominated by lymphocytes and plasma cells and occasionally accompanied by interstitial fibrosis in 15 foxes. In addition, single cases of granulomatous nephritis (n = 2), renal tubular mineralisation (n = 3), cystitis (n = 3) and endometritis (n = 2) were diagnosed.

#### Musculoskeletal system

Skeletal lesions were rare. Old fractures with callus formation, present in ribs and thoracic vertebra were seen in four foxes (5%). One aged fox showed spondyloses of the lumbar spinal column and one severe arthrosis of the stifle joint. In one stone marten, chronic gonarthrosis was present, accompanied by atrophy of the musculature of the affected limb. Mild myofibrillar degeneration was present in the skeletal musculature of one fox and one stone marten. Two animals showed intramuscular protozoal cysts, morphologically consistent with *Sarcocystis* cysts.

#### Integument

Four foxes and three stone martens showed macroscopic skin lesions. These included an old wound at the limb of one fox with demarcating granulation tissue proliferation and suppurative inflammation as well as a severe, chronic suppurative dermatitis at the ear base of another fox. Two animals showed single focal skin ulcerations on the nasal ridge (Fig 3). The most frequent finding of the facial skin was multifocal, mild, chronic lymphohistiocytic dermatitis which was present in nine foxes and one stone marten. In one fox, periocular dermatitis with intralesional fungal organisms was demonstrated. Furthermore, five foxes and four stone martens showed macroscopical evidence of ectoparasites, including nymphs or adults of Ixodid ticks, and one fox showed a moderate flea infestation.

#### Miscellaneous lesions

Additional findings included a mild, suppurative and follicular conjunctivitis in three foxes and one stone marten. Moreover, a thyroid adenoma (n = 1), a cataract (n = 2), a nodular hyperplasia of the adrenal cortex (n = 1), lymphoplasmacytic infiltrations of the pancreas (n = 1) and lymphadenitis (n = 7) were seen in foxes. A mild esophagitis (n = 1), lymphadenitis (n = 1) and a mild adrenalitis (n = 1) were found in stone martens.

### Viral metagenomics and RT-PCR

In two foxes, either one or two reads were detected that showed closest similarity to anelloviruses, while in the brain tissue samples collected from the other three foxes and the stone marten no reads were detected that had the closest similarity to viral sequences (S2 Table).

### Investigation for fox circovirus DNA by RT-qPCR in foxes with lymphohistiocytic encephalitis

FFPE tissue samples of the foxes suffering from lymphohisticytic encephalitis (n = 9) were tested for circovirus DNA using RT-qPCR and specific primers. Fox circovirus DNA was not detected in tested animals, while canine GAPDH was present in all tested fox samples with cycle threshold (Ct)-values ranging from 21.25 to 24.61, as well as PCV-2 specific DNA in the pig serving as additional positive control (Ct-value: 16.83).

### Serum neutralisation test

30 animals (16 foxes, 12 stone martens and two raccoon dogs) were tested for the presence of CDV antigens. Non-infected Vero cells were used as negative controls in a dilution of 1:10. Virus titers over 1:20 were interpreted as positive after five days of incubation [64]. The results showed that only one fox had a virus-neutralisation antibody titer of 1:160.

## Discussion

The aim of the present study was to identify ongoing infectious disease processes in wildlife predators in Northern Germany and their potential transmission to other animal species and humans. The major findings included (i) 46 cases (45.6% foxes, 35.3% stone martens and 40% raccoon dogs) of endoparasitism including agents with high zoonotic potential; (ii) 17 cases (11.4% foxes, 23.5% stone martens and 30% raccoon dogs) of encephalitis and (iii) 28 cases (24% foxes, 17.6% stone martens and 60% raccoon dogs) with pneumonia of unknown origin.

One important feature of the present study refers to the number of parasitic infestations, which were detected in a considerable number of animals (36/79 foxes, 6/10 raccoon dogs and 4/17 stone martens). No correlation could be made between the health status of the animals and the severity of parasitic infestation. Histologically, adult stages of both *Taenia* sp. and *Toxocara* sp. in the intestines, and *Alaria* sp. mesocercariae in the lung parenchyma, were detected. These parasites are known to bear a zoonotic potential for humans [2, 65–68]. Cestodes in the intestine of nine animals, indicates that wild carnivores play a considerable role in shedding *Echinococcus* spp. eggs. This further leads to a persistent source of infection not only for rodents but also for humans as incidental hosts [69, 70].

Even though the annual incidence of human alveolar echinococcosis in Germany seemed to be low in 2006 [71, 72], the growing red fox population as well as the increasing urbanisation of habitats bear a high potential for zoonotic spread to humans [8, 70]. Trematodes consistent with *Alaria* sp. further point to other, less widespread but nonetheless threatening zoonotic parasites, which might be spread by wildlife predators and invertebrates as intermediate hosts. In paratenic hosts, such as wild boars, *Alaria* sp. mesocercariae may be found in adipose, muscular or connective tissue, from which they can be ingested by humans eating raw or inadequately cooked meat [66]. Few reports concerning humans exist, where the disease occurs due to migrating larvae which penetrate the intestinal wall and manifest in various clinical signs [66].

Furthermore, there has been a considerable number of animals with encephalitis of undetectable cause. A detailed neurological investigation was not performed and neurological dysfunctions or behavioural impairment cannot be ruled out. However, severe neurological impairment or clinical signs indicating CNS disease were not detected in trapped animals or reported by hunters. The morphology of these CNS lesions pointed clearly to a viral etiology. Nevertheless, no causative agents were detected in any of the cases despite undertaking extensive immunohistochemical investigations and advanced molecular analysis to detect commonly known as well as new viral pathogens in carnivores. Of interest, analysis of the brain tissues of six animals with meningo-encephalitis by viral metagenomics revealed very low numbers, with the closest similarity to members of the *Anelloviridae* in two samples, while no significant viral reads were detected in the other four brain tissues samples. These findings indicate that it is unlikely that a high virus load was present in the tested tissue [73]. However, it cannot be ruled out that an infection with a low viral load was present in the tested tissues, which could be detected with other, novel NGS methods. This might be a promising approach for further investigations. The evidence of readings with similarities to *Anelloviridae* is of interest, as they have a high prevalence in the human population and are associated with hepatitis or pathological conditions of the respiratory tract or immune system. Namely, there exist some reports highlighting the disease-inducing potential of *Anelloviridae* [74–77]. Furthermore, they have also been detected in different animal species, thus leading to the hypothesis that they can be transmitted to humans and *vice versa* [77]. However, regarding their ubiquitous distribution and their unresolved pathogenicity, it is more likely that their detection only represents a secondary, epiphenomal response [74–77]. In conclusion, the detailed role of anelloviruses in the present investigated cases remains unclear, and it is not likely that they are the causative agents of the observed encephalitis.

One of the most prevalent causes for encephalitis in foxes is canine distemper virus infection [78–80]. However, immunohistochemistry revealed lacking CDV antigen expression as well as lacking immunoreactivity for other viral antigens, namely Borna virus, feline leukaemia virus (FeLV), parvovirus, tick-borne encephalitis (TBE) virus, influenza A virus and canine adenovirus (CAV1). The positivity in the virus-neutralisation test in one animal indicates that CDV infection may occur in foxes but seems to be of low prevalence in the investigated population and region. This might partially be explained by a cyclic course of infection with episodes of disease outbreaks and disease-free intervals [81, 82]. Regarding the coincidence of the influenza-outbreak in seals [34] and the time of hunting and trapping of predators living in close vicinity to the coast, influenza-A-virus was regarded as a possible cause for inflammatory lesions, especially in the respiratory tract. However, no immuno-positivity for avian influence A virus could be demonstrated. This might be explained either by low contact with infected animals/ carcasses, low infection pressure, or as a consequence of low susceptibility of the investigated population to the pathogen in question.

Recent investigations of foxes in central and southern parts of Germany (Bavaria, Baden-Wuerttemberg and Hesse) with special emphasis on Borna disease virus, revealed 10/ 16 cases with encephalitis lacking specific pathogens [83]. Similarly, recent studies in dogs and cats indicated that the majority of cases with encephalitis remained etiologically undetermined [48].

Nonetheless, in all these cases, similarly to the present study, negative results do not rule out that viral infection played a role in the development as a trigger of CNS lesions (e.g. infection may have occurred some time ago and immune response cleared the infection at the time of investigation. Another possibility is that infections might have triggered an autoimmune disease, molecular mimicry and/or epitope spread, resulting in a form of late sequel of virus infection [48]. This phenomenon might explain the high number of cases with encephalitis lacking a detectable infectious cause in the present study.

An etiological differential diagnosis for non-suppurative or granulomatous inflammation within the CNS are specific bacteria or several protozoal or fungal organisms, most importantly *Listeria monocytogenes*, *Neospora caninum*, *Toxoplasma gondii* and *Enzephalitozoon cuniculi* [48, 84–86]. All animals were immunohistochemically negative for *Listeria monocytogenes*, *Neospora caninum* and *Toxoplasma gondii*. Also, no intralesional protozoal structures were identified within the lesions. This could either be explained by the fact that the mentioned agents are not endemic in this area or that they are not present in the tissue any longer. Interestingly, in bordering areas of northern Germany the prevalence for specific agents like *Toxoplasma gondii* or *Encephalitozoon cuniculi* in the red fox population is high [84, 86, 87]. This might thus represent an interesting topic for further specific studies dealing with bacterial and protozoal infections in wild predators and their potentially infected intermediate hosts in this region. [84, 86, 87]

Another, non-infection-related mechanism causing non-suppurative encephalitis has recently been described in the deceased captive polar bear Knut, where antibodies against the N-methyl-D-aspartate (NMDA) receptor were demonstrated in the cerebrospinal fluid, causing autoimmune encephalitis [88]. This phenomenon might also be the cause of some types of encephalitis of unknown etiology in the investigated cases. However, further examinations are required to determine possible non-viral causes of encephalitis in wild carnivores.

Regarding the general health status of the animals examined in this study, the most prevalent findings were chronic interstitial nephritis, myocardial changes, skin lesions and findings in the gastrointestinal tract including the liver. Overall, these types of lesions excluding the changes in the kidneys were characterised by a low degree of severity and must be considered as incidental findings with no clinical significance at the time of investigation. Chronic interstitial nephritis is known among wildlife species and often seen within domestic carnivores. The etiology remains unclear in most cases and it would seem that protein-rich nutrition possibly favours the condition. Moreover, acquired infectious (such as leptospirosis) or non-infectious (such as autoimmune) diseases or congenital factors might be involved [45, 89, 90]. Mild bacterial or viral infections are primarily suspected of causing the inflammation. Skin lesions appeared to be mostly related to minor traumatic injury with secondary bacterial infection. Interestingly, detectable ectoparasitism was generally low. Only single animals displayed evidence of tick nymph infestation; single adult ticks and one animal showed a light infestation of fleas. Gastrointestinal tract lesions were mild in most cases and presumably related to parasitic infection. A surprisingly high number of stone martens displayed mild hepatitis of unknown cause. Further investigations into the underlying cause might be of interest in future studies.

In line with the “One-Health” notion, this study highlights the importance of periodical investigations on the health status of wild animals living in close contact to humans and domestic animals. The results provide an update on the health status of wild foxes, stone martens and raccoon dogs in Northern Germany and highlight the persistent risk of transmissible, infectious zoonotic agents which might be spread among wild and domestic animals as well as humans. The high number of etiologically undetermined encephalitis and inflammatory processes in several cases underline the importance of further studies on the detection of novel infectious diseases and immune-mediated inflammatory diseases.

## Supporting information

S1 Table Examined animals: Animal number, laboratory identification number, date of necropsy, species, age (determined by teeth), gender, cause of death and main macroscopical and histological lesions. (DOCX)

S2 Table Overview of results of viral metagenomics performed on brain tissues from foxes and a stone marten. (DOCX)
