# Supplementary material for: Pathological findings in the red fox (Vulpes vulpes), stone marten (Martes foina) and raccoon dog (Nyctereutes procyonoides), with special emphasis on infectious and zoonotic agents in Northern Germany
Source: PLoS One. 2017 Apr 11;12(4):e0175469. doi: 10.1371/journal.pone.0175469 (PMC5388480; doi:10.1371/journal.pone.0175469)
Supplement: S1 Table — (DOCX) [file pone.0175469.s001.docx]

**Supporting information**

**S1 Table: Examined animals: animal number, laboratory identification number, date of necropsy, species, age (determined by teeth), gender, cause of death and main macroscopical and histological lesions.** SNT, Serum neutralisation test for canine distemper virus; IN, interstitial nephritis; IP, interstitial pneumonia; GP, granulomatous pneumonia; E, encephalitis; ME, meningoencephalitis; H, hepatitis.

| **Animal**  **number** | **Laboratory identification number** | **Date of necropsy** | **Species** | **Age** | **Male/female** | **Cause of death** | **Main macroscopical and histological lesions** |
| --- | --- | --- | --- | --- | --- | --- | --- |
| 1 | V/812/13 | 04.11.13 | Red fox | <1year | Male | euthanasia | IN; old rib fracture; lymphohistiocytic to fibrino-suppurative dermatitis with granulation tissue; anal sac (nematode stages); lymphadenitis; intranasal (nematode stages); lymphohistiocytic to eosinophilic rhinitis; lung (nematode stages); suppurative-eosinophilic pneumonia; small intestine (trematode stages); |
| 2 | V/813/13 | 04.11.13 | Red fox | Adult | Female | euthanasia | lymphohistiocytic ME, aortic aneurysm, IN; ticks; fleas; large intestine (cestode stages); cystitis; SNT (1:160); |
| 3 | V/826/13 | 15.11.13 | Red fox | Adult | Male | euthanasia | spondyloses; lymphohistiocytic glossitis; lymphohistiocytic to eosinophilic rhinitis (nematode stages); |
| 4 | V/840/13 | 19.11.13 | Red fox | Adult | Male | euthanasia | IN; ulcerative dermatitis; SNT (<1:10); |
| 5 | V/865/13 | 29.11.13 | Red fox | Adult | Male | euthanasia | lymphohistiocytic ME predominantly in the cerebellum (vacuolisation); IN; aortic dextroposition; |
| 6 | V/866/13 | 29.11.13 | Red fox | Adult | Male | euthanasia | uremia; stomach (nematode stages); lymphohistiocytic to eosinophilic rhinitis (nematode stages); |
| 7 | V/3/14 | 03.01.14 | Red fox | Adult | Female | euthanasia | IN; ulcerative dermatitis; large intestine (cestode and nematode stages); |
| 8 | V/17/14 | 08.01.14 | Red fox | Adult | Female | euthanasia | suppurative dermatitis; tonsillitis; esophagitis; |
| 9 | V/20/14 | 09.01.14 | Red fox | Adult | Female | euthanasia, | small and large intestine (nematode stages); |
| 10 | V/21/14 | 09.01.14 | Red fox | Adult | Male | euthanasia | tooth loss; alveolitis; thyroid adenoma; SNT (<1:10); |
| 11 | V/37/14 | 16.01.14 | Red fox | Adult | Female | euthanasia | IN; SNT (<1:10); |
| 12 | V/57/14 | 30.01.14 | Red fox | Adult | Male | shot | lymphohistiocytic H; SNT (<1:10); |
| 13 | V/59/14 | 04.02.14 | Red fox | Adult | Female | euthanasia | myocardial mineralisation; granulomatous dermatitis; SNT (<1:10); |
| 14 | V/208/14 | 26.02.14 | Red fox | Adult | Male | euthanasia | lymphoplasmacytic E, old fracture (forelimb); lymphoplasmahistiocytic dermatitis; anal sac (nematode stages); large intestine (cestode stages); SNT(<1:10); |
| 15 | V/213/14 | 27.02.14 | Red fox | Adult | Male | euthanasia | trachea (nematode stages); lymphohistiocytic to suppurative H; large intestine (cestode stages); SNT (<1:10); |
| 16 | V/302/14 | 08.04.14 | Red fox | Adult | Female | shot | lymphohistiocytic E; |
| 17 | V/303/14 | 08.04.14 | Red fox | Adult | Female | shot | lymphocytic E; IN; anal sac (nematode stages); lymphoplasmahistiocytic to suppurative rhinitis and tracheitis (nematode stages); lymphohistiocytic H; |
| 18 | V/304/14 | 10.04.14 | Red fox | Adult | Female | shot | lymphoplasmahistiocytic E; |
| 19 | V/305/14 | 10.04.14 | Red fox | Adult | Male | shot | no significant lesions; |
| 20 | S/772/14 | 30.07.14 | Red fox | Juvenile | Female | shot | eosinophilic enteritis; small intestine (cestode stages); |
| 21 | S/775/14 | 31.07.14 | Red fox | Adult | Male | shot | no significant lesions; |
| 22 | S/831/14 | 13.08.14 | Red fox | Adult | Male | shot | IN; lymphoplasmacellular H; |
| 23 | S/880/14 | 26.08.14 | Red fox | Juvenile | Female | shot | IN; SNT (<1:10); |
| 24 | S/895/14 | 28.08.14 | Red fox | Juvenile | Male | shot | small intestine (nematode and cestode stages); |
| 25 | S/896/14 | 28.08.14 | Red fox | Juvenile | Male | shot | no significant lesions; |
| 26 | V/621/14 | 30.10.14 | Red fox | Adult | Male | euthanasia | plexus choroiditis; small and large intestine (cestode stages); SNT (<1:10); |
| 27 | V/664/14 | 26.11.14 | Red fox | Adult | Female | euthanasia | lymphadenitis; small intestine (nematode stages); |
| 28 | V/665/14 | 26.11.14 | Red fox | Adult | Male | shot | kidney: tubular mineralisation; |
| 29 | V/666/14 | 26.11.14 | Red fox | Adult | Female | shot | myocarditis; IP; |
| 30 | V/670/14 | 27.11.14 | Red fox | Adult | Female | euthanasia | IN; ulcerative dermatitis; anal sac (nematode stages); SNT (<1:10); |
| 31 | V/671/14 | 09.12.14 | Red fox | Adult | Male | shot | IP; cystitis; |
| 32 | V/672/14 | 09.12.14 | Red fox | Adult | Female | euthanasia | no significant lesions; |
| 33 | V/676/14 | 11.12.14 | Red fox | Adult | Female | shot | atrioventricular endocardiosis; suppurative inflammation -anal sac; |
| 34 | V/677/14 | 11.12.14 | Red fox | Adult | Male | shot | GP; coat: tick stages; |
| 35 | V/678/14 | 11.12.14 | Red fox | Adult | Female | shot | kidney: tubular mineralisation; |
| 36 | V/679/14 | 11.12.14 | Red fox | Adult | Male | shot | lung: osseous metaplasia; |
| 37 | V/687/14 | 17.12.14 | Red fox | Adult | Female | euthanasia | IN; cataract; SNT (<1:10); |
| 38 | V/688/14 | 17.12.14 | Red fox | Adult | Female | shot | vacuoles in the CNS; |
| 39 | V/689/14 | 17.12.14 | Red fox | Adult | Female | shot | lymphocytic ME; |
| 40 | V/18/15 | 15.01.15 | Red fox | Adult | Female | euthanasia | anal sac: lymphohistiocytic inflammation (nematode stages); SNT (<1:10); |
| 41 | V/35/15 | 22.01.15 | Red fox | Adult | Female | shot | anal sac: lymphohistiocytic inflammation; tonsillitis; |
| 42 | V/36/15 | 22.01.15 | Red fox | Adult | Female | shot | lymphocytic ME; granulomatous glossitis; IP; lymphohistiocytic H; |
| 43 | V/37/15 | 22.01.15 | Red fox | Adult | Male | shot | anal sac: lymphohistiocytic to suppurative inflammation; tonsillitis; |
| 44 | V/38/15 | 22.01.15 | Red fox | Adult | Male | shot | anal sac: lymphohistiocytic inflammation; |
| 45 | V/106/15 | 29.01.15 | Red fox | Adult | Male | shot | IN; E (brain stem); anal sac: suppurative inflammation; |
| 46 | V/107/15 | 29.01.15 | Red fox | Adult | Male | shot | granulomatous nephritis; anal sac: lymphohistiocytic inflammation; |
| 47 | V/108/15 | 29.01.15 | Red fox | Adult | Female | shot | no significant lesions; |
| 48 | V/124/15 | 05.02.15 | Red fox | Adult | Female | euthanasia | conjunctivitis; SNT (<1:10); |
| 49 | V/175/15 | 25.02.15 | Red fox | Adult | Male | shot | lymphohistiocytic dermatitis; |
| 50 | V/260/15 | 10.04.15 | Red fox | Adult | Female | shot | lymphohistiocytic glossitis; |
| 51 | V/261/15 | 10.04.15 | Red fox | Adult | Female | shot | anal sac: suppurative inflammation; conjunctivitis; |
| 52 | V/262/15 | 10.04.15 | Red fox | Adult | Female | shot | unilateral, IN; lipid pneumonia; |
| 53 | S/430/15 | 21.04.15 | Red fox | Adult | Female | shot | suppurative endometritis; ulcerative gastritis; |
| 54 | S/434/15 | 21.04.15 | Red fox | Juvenile | Female | shot | no significant lesions; |
| 55 | S/435/15 | 21.04.15 | Red fox | Juvenile | Male | shot | no significant lesions; |
| 56 | S/436/15 | 21.04.15 | Red fox | Juvenile | Female | shot | no significant lesions; |
| 57 | S/514/15 | 12.05.15 | Red fox | Juvenile | Male | shot | pneumonia, dehydration; |
| 58 | S/515/15 | 12.05.15 | Red fox | Juvenile | Male | shot | no significant lesions; |
| 59 | V/862/15 | 20.08.15 | Red fox | Adult | Male | shot | bronchitis; |
| 60 | V/863/15 | 20.08.15 | Red fox | Adult | Male | shot | GP; lung (nematode stages); liver necrosis; tonsillitis; |
| 61 | V/953/15 | 03.09.15 | Red fox | Juvenile | Female | shot | catarrhal enteritis; |
| 62 | V/954/15 | 03.09.15 | Red fox | Juvenile | Female | shot | pneumonia; suppurative to necrotising H; |
| 63 | V/955/15 | 03.09.15 | Red fox | Juvenile | Female | shot | no significant lesions; |
| 64 | V/956/15 | 03.09.15 | Red fox | Juvenile | Female | shot | no significant lesions; |
| 65 | V/1155/15 | 26.10.15 | Red fox | Adult | Male | shot | bronchitis; |
| 66 | V/1156/15 | 26.10.15 | Red fox | Juvenile | Female | shot | pneumonia, IN; necrotising H; gliosis; meninx mineralisation; |
| 67 | V/1166/15 | 28.10.15 | Red fox | Adult | Male | shot | enteritis; IP; anal sac (nematode stages); |
| 68 | V/1221/15 | 25.11.15 | Red fox | Adult | Female | shot | pneumonia; trachea (nematode egg); |
| 69 | V/1223/15 | 25.11.16 | Red fox | Adult | Female | shot | no significant lesions; |
| 70 | V/1236/15 | 27.11.15 | Red fox | Adult | Female | shot | no significant lesions; |
| 71 | V/1238/15 | 27.11.15 | Red fox | Adult | Male | shot | bronchitis; IN; |
| 72 | V/1239/15 | 27.11.15 | Red fox | Adult | Female | shot | no significant lesions; |
| 73 | V/48/16 | 11.01.16 | Red fox | Adult | Male | shot | IN and mesangial GN; |
| 74 | V/49/16 | 11.01.16 | Red fox | Adult | Female | shot | no significant lesions; |
| 75 | V/50/16 | 11.01.16 | Red fox | Adult | Female | shot | no significant lesions; |
| 76 | V/51/16 | 11.01.16 | Red fox | Adult | Male | shot | pneumonia, arthrosis of the stifle joint; lymphohistiocytic to necrotising H; cystitis; |
| 77 | S/94/16 | 21.01.16 | Red fox | Adult | Female | shot | lymphohistiocytic H; large intestine (trematode stages); gliosis; |
| 78 | S/96/16 | 21.01.16 | Red fox | Adult | Male | shot | nodular hyperplasia of the liver; anal sac (nematode stages); large intestine (nematode stages); |
| 79 | S/102/16 | 21.01.16 | Red fox | Adult | Male | shot | suppurative bronchitis; enteritis; |
| 80 | V/853/13 | 20.11.13 | Stone marten | Adult | Female | euthanasia | suppurative dermatitis; lymphohistiocytic to suppurative H; SNT (<1:10); |
| 81 | V/857/13 | 22.11.13 | Stone marten | Adult | Male | euthanasia | gonarthrosis; histiocytic lymphadenitis; myocard (sarcosporidian cysts); lymphohistiocytic H; SNT (<1:10); |
| 82 | V/862/13 | 26.11.13 | Stone marten | Adult | Male | euthanasia | no significant lesions; SNT (<1:10); |
| 83 | V/863/13 | 27.11.13 | Stone marten | Adult | Female | euthanasia | GP; SNT (<1:10); lymphohistiocytic H; |
| 84 | V/864/13 | 29.11.13 | Stone marten | Juvenile | Female | euthanasia | no significant lesions; |
| 85 | V/870/13 | 03.12.13 | Stone marten | Juvenile | Male | euthanasia | lymphohistiocytic leukoencephalitis; ulcerative to suppurative dermatitis; lympho-histiozytic H; SNT (<1:10); |
| 86 | V/974/13 | 17.12.13 | Stone marten | Adult | Male | euthanasia | GP; liver necrosis; IN; |
| 87 | V/36/14 | 16.01.14 | Stone marten | Juvenile | Female | euthanasia | no significant lesions; SNT (<1:10); |
| 88 | V/38/14 | 21.01.14 | Stone marten | Adult | Female | euthanasia | no significant lesions; anal sac (nematode stages); SNT (<1:10); |
| 89 | V/56/14 | 29.01.14 | Stone marten | Adult | Female | euthanasia | lymphocytic E; tick nymphs; anal sac (nematode stages); sarcosporidian cyst; lymphohistiocytic to suppurative H; SNT (<1:10); |
| 90 | V/301/14 | 08.04.14 | Stone marten | Adult | Female | shot | IN; |
| 91 | V/120/15 | 03.02.15 | Stone marten | Adult | Male | euthanasia | ME (cervical spinal cord); tick nymphs; dermatitis; lymphohistiocytic H; SNT (<1:10); |
| 92 | V/121/15 | 03.02.15 | Stone marten | Adult | Female | euthanasia | lymphoplasmacytic E; anal sac (nematode stages); |
| 93 | V/125/15 | 05.02.15 | Stone marten | Adult | Male | euthanasia | IP; anal sac: lymphohistiocytic to suppurative inflammation (nematode stages); SNT (<1:10); |
| 94 | V/195/15 | 18.03.15 | Stone marten | Adult | Female | euthanasia | anal sac: lymphohistiocytic inflammation; SNT (<1:10); |
| 95 | V/1222/15 | 28.10.15 | Stone marten | Adult | Female | shot | no significant lesions; |
| 96 | V/1237/15 | 27.11.15 | Stone marten | Adult | Female | shot | no significant lesions; |
| 97 | V/690/14 | 17.2.14 | Raccoon dog | Adult | Female | shot | GP; granulomatous glossitis; |
| 98 | V/109/15 | 29.01.15 | Raccoon dog | Adult | Male | shot | lymphohistiocytic ME; granulomatous glossitis; |
| 99 | V/122/15 | 03.02.15 | Raccoon dog | Adult | Male | shot | granulomatous E; GP (nematode stages); small intestine (cestodes and trematode stages); |
| 100 | V/1163/15 | 26.10.15 | Raccoon dog | Juvenile | Female | shot | GP; IN; lung (nemtodes); necrotising H; |
| 101 | V/1165/15 | 28.10.15 | Raccoon dog | Adult | Male | found dead | lymphoplasmacytic meningitis; suppurative pneumonia; SNT (<1:10); |
| 102 | V/1167/15 | 28.10.15 | Raccoon dog | Adult | Female | shot | myocarditis; pneumonia; IN; |
| 103 | V/1168/15 | 28.10.15 | Raccoon dog | Juvenile | Female | found dead | suppurative H; anal sac: suppurative inflammation; lung (nematode stages); SNT (<1:10); |
| 104 | V/1224/15 | 25.11.15 | Raccoon dog | Adult | Female | shot | GP; large intestine (cestode stages); |
| 105 | S/95/16 | 21.01.16 | Raccoon dog | Adult | Male | shot | suppurative pneumonia; GN; |
| 106 | S/103/16 | 21.01.16 | Raccoon dog | Adult | Female | shot | GP; gastritis; |
