# Supplementary material for: Pathological findings in the red fox (Vulpes vulpes), stone marten (Martes foina) and raccoon dog (Nyctereutes procyonoides), with special emphasis on infectious and zoonotic agents in Northern Germany
Source: PLoS One. 2017 Apr 11;12(4):e0175469. doi: 10.1371/journal.pone.0175469 (PMC5388480; doi:10.1371/journal.pone.0175469)
Supplement: S2 Table — (DOCX) [file pone.0175469.s002.docx]

**Supporting information**

**S2 Table**. **Overview of results of viral metagenomics performed on brain tissues from foxes and a stone marten.**

| Animal Number | Species | Number of reads analyzed | Number of reads most closely related to viral sequences (virus family) |
| --- | --- | --- | --- |
| 2 | Red fox  (*Vulpes vulpes*) | 22131 | 1 (*Anelloviridae)* |
| 14 | Red fox  (*Vulpes vulpes*) | 12985 | None |
| 16 | Red fox  (*Vulpes vulpes*) | 19604 | None |
| 17 | Red fox  (*Vulpes vulpes*) | 14157 | 2 (*Anelloviridae*) |
| 18 | Red fox  (*Vulpes vulpes*) | 4862 | None |
| 85 | Stone marten  (*Martes foina*) | 21313 | None |
